# Genome Report—A Genome Sequence Analysis of the RB51 Strain of *Brucella abortus* in the Context of Its Vaccine Properties

**DOI:** 10.1534/g3.119.400964

**Published:** 2020-02-28

**Authors:** Betsy Bricker, Nalin Goonesekere, Darrell Bayles, David Alt, Steven Olsen, Catherine Vrentas

**Affiliations:** * Infectious Bacterial Diseases Research Unit, National Animal Disease Center, Agricultural Research Service, U.S. Department of Agriculture, Ames, IA, 50010; † Department of Chemistry and Biochemistry, University of Northern Iowa, Cedar Falls, IA, 50614

**Keywords:** Brucella, RB51, *B. abortus*, Brucellosis, vaccine sequence

## Abstract

The RB51 vaccine strain of *Brucella abortus*, which confers safe and effective protection of cattle from *B. abortus* infection, was originally generated via serial passage of *B. abortus* 2308 to generate spontaneous, attenuating mutations. While some of these mutations have been previously characterized, such as an insertional mutation in the *wboA* gene that contributes to the rough phenotype of the strain, a comprehensive annotation of genetic differences between RB51 and *B. abortus* 2308 genomes has not yet been published. Here, the whole genome sequence of the RB51 vaccine strain is compared against two available 2308 parent sequences, with all observed single nucleotide polymorphisms, insertions, and deletions presented. Mutations of interest for future characterization in vaccine development, such as mutations in *eipA* and *narJ* genes in RB51, were identified. Additionally, protein homology modeling was utilized to provide *in silico* support for the hypothesis that the RB51 *capD* mutation is the second contributing mutation to the rough phenotype of RB51, likely explaining the inability of *wboA*-complemented strains of RB51 to revert to a smooth phenotype.

Brucellosis, a chronic and debilitating bacterial disease in humans, is spread zoonotically from infected ruminant species like goats and cattle, in which the disease causes reproductive dysfunction and abortion in pregnant animals (reviewed by de Figueiredo *et al.* 2015). Multiple species of *Brucella* are infectious to humans; *Brucella abortus* is often found in cattle, *Brucella suis* in pigs and feral swine, and *Brucella melitensis* in sheep and goats.

Vaccination of young animals is an important strategy for protection of both animals and humans against infection with *Brucella* species. Multiple live, attenuated vaccines have been developed for protection of livestock against *Brucella abortus*, including RB51 (Schurig *et al.* 1991; Cheville *et al.* 1993), used in the U.S. in cattle herds. However, development of vaccines against *Brucella* species that are both safe and effective is made challenging by the intracellular nature of the bacterium.

Investigation of the characteristics of the RB51 *B. abortus* vaccine strain is of importance for development of future *B. melitensis* vaccines, due to its ability to elicit a strong protective, cell-based immune response without causing disease in livestock (reviewed by Schurig *et al.* 2002). While *B. melitensis* is highly virulent in humans and causes zoonotic transmission and persistent infection (reviewed by Atluri *et al.* 2011), no vaccines against *B. melitensis* are currently approved in the U.S. as safe and effective for use in humans. RB51 was derived from *B. abortus* strain 2308 by serial passage, and exhibits a rough phenotype in its cell envelope (Schurig *et al.* 1991). It is well-known that an IS711 element-caused disruption is present in the *wboA* gene of RB51; *wboA* encodes a glycosyltransferase necessary for lipopolysaccharide (LPS) biosynthesis, and this disruption is at least partially responsible for the rough morphology of RB51 (Vemulapalli *et al.* 1999). However, complementation of the defective gene with the wild-type *wboA* allele did not restore a smooth phenotype or restore virulence to RB51, so additional factors appear to contribute to the strain phenotype (Vemulapalli *et al.* 2000). Not all rough strains of *Brucella* confer the protective properties of RB51 (*i.e.*, Elzer *et al.* 1998), indicating that there are genetic changes specific to RB51 that contribute to its beneficial vaccine properties.

While sequencing of the RB51 genome has been attempted, previous sequences were either not closed to completion, or are disparate from expected allele sequences at the *wboA* gene locus (Ma *et al.* 2014; Table S1; Figure S1). Additionally, a genome-wide (complete) comparison of RB51 to the parent *B. abortus* 2308 strain has not yet been published. Therefore, to provide a complete picture of genetic changes that may contribute to RB51 properties, we present here a sequence of the RB51 vaccine strain closed to completion at the National Animal Disease Center, assess DNA mutations relative to the 2308 genome sequence, and compare findings to previous studies. Using protein modeling, we then predict the impact of mutations of particular interest for use in future vaccine development.

## Materials & Methods

### DNA isolation and purification

Genomic DNA was extracted as previously described (Halling *et al.* 2005) from approximately 1 X 10^10^ methanol-killed *Brucella abortus* RB51 (sub-strain ARS-1). After the second precipitation, the spooled DNA was washed for several days in periodic changes of 80% ethanol. The purified DNA was stored in 80% ethanol. As needed, precipitated flakes of DNA were removed and re-solubilized in 10 mM sodium hydroxide. When fully rehydrated, the pH was adjusted to 7.5 in the presence of 10 mM HEPES buffer, and stored at 4°.

### DNA sequencing

The RB51 genomic DNA was sequenced by two methods. The first pass was done by Sanger sequencing of purified plasmid DNA from a shotgun library created from the RB51 genomic DNA. The DNA was sequenced on an ABI-PRISM DNA Sequencer, Model 3700, following treatment of the plasmid DNA with BigDye terminator nucleotides (ABI_PRISM BigDye Terminator Cycle Sequencing Kit ver. 3.0; Applied Biosystems, Foster City, CA, USA) as directed by the manufacturer. A second round of sequencing was done by pyrosequencing with the high-throughput Roche GS FLX Sequencing System per manufacturer’s directions (454 Life Sciences- A Roche company, Branford, CT, USA). Briefly, the RB51 purified DNA was sheared by nebulization to a range of approximately 500 to 700 base pairs. A single-strand template (sstDNA) shotgun library was created from the sheared fragments as directed in the GS FLX Standard Library Preparation Kit (Roche Applied Science, Indianapolis, IN, USA). DNA concentrations were measured with an Agilent 2100 Bioanalyzer (Agilent Technologies, Palo Alto, CA, USA). The size range of the sheared DNA was analyzed on a DNA-7500 lab chip, while the concentration of the sstDNA library was determined on the Pico 6000 LabChip. The RB51-sstDNA library was amplified by emulsion-based clonal amplification (emPCR) as directed by the Shotgun GS FLX Standard emPCR kit (Roche Applied Science) manual. The DNA-bound beads were loaded onto a Pico Titer plate and sequenced with the GS LR70 Sequencing Kit per manufacturer’s instructions.

### Sequence assembly and annotation

Image processing of the raw data and signal processing (base calling) were performed using the Roche GS Run Processor pipeline according to the manufacturer’s recommendations. Assembly of sequence data into contigs was completed with Phred/Phrap and Consed software programs. Phred was used for base calling of the Sanger sequence reads, while Phrap was used to assemble both the Sanger and the pyrosequencing data. Consed was used for the graphical assembly and final editing. The annotated sequences were prepared for submission to GenBank with the IMG/ER software program (Joint Genomic Initiative; http://img.jgi.doe.gov/er; Markowitz *et al.* 2009). As needed, the annotated sequence was viewed with the Artemis sequence viewer and annotation tool (Genome Research Limited; http://www.sanger.ac.uk/Software/Artemis; Mural 2000). Results were compared manually with ACT, a DNA comparison viewer, part of the Artemis package. Genes were called using Glimmer (Salzberg *et al.* 1998), and compared to homologs using BLAST (Altschul *et al.* 1990). Single nucleotide polymorphisms (SNPs) were identified with MUMmer (http://www.tigr.org/software/mummer; Kurtz *et al.* 2004) and sequence alignment with Mauve (http://gel.ahabs.wisc.edu/mauve; Darling *et al.* 2004).

### Protein modeling

Computational modeling of the C-terminal domain of CapD (residues 261 - 585) was performed using the program I-TASSER (Roy *et al.* 2010; https://zhanglab.ccmb.med.umich.edu/I-TASSER/). I-TASSER has been the top-ranked server in the last few Critical Assessment of Techniques for Protein Structure Prediction (CASP 7-10) competitions for homology modeling and threading. The C-terminal domain structure of PgIF (PDB ID: 5BJU), which had a sequence identity of 41% with the C-terminal domain of CapD, was used as the template. The five structural models normally returned by the program was collapsed to one, indicating the generation of a high-quality model. This is confirmed by the high C-score (1.23) for the model (the C-score describes the quality of a predicted model, and ranges between -5 and 2). When the model was superposed with the C-terminal domain of PgIF, the only significant deviations occurred in two loop regions located far away from the active site of PgIF (Figure S2). PDB files were visualized using PyMol software.

### Data availability

The genome information is publicly available on NCBI GenBank (“whole genome sequence of *Brucella abortus* RB51, ARS-1 isolate”) under the BioProject accession PRJNA573988 and BioSample accession SAMN12837785. Strains are available upon request to the USDA ARS National Animal Disease Center. Table S1 provides an overview of features of RB51 genomes, and Table S2 provides an overview of features of this RB51 genome as compared to other published *B. abortus* genomes. Table S3 describes sequence analysis of LPS synthesis genes from RB51. Figure S1 depicts an alignment of the *wboA* sequences from NZ_AQIE00000000.1 and *B. abortus* 2308. Figures S2 and S4 depict alignment of the protein sequences of CapD and PglF. Figure S3 depicts superposition of structures of the PglF protein and the model of the CapD protein. Figure S5 depicts superposition of structures of the *S. aureus* CapE protein and the model of the CapD protein. Supplemental material available at figshare: https://doi.org/10.25387/g3.11864358.

## Results

### RB51 gene sequence as compared to 2308 parent strain

We assembled to closure and annotated the sequence of both chromosomes of the *B. abortus* RB51 genome (genome size and characteristics provided in Table S2), and then compared our assembled genome with that of two GenBank submissions for *B. abortus* 2308 (Table 1). The first, designated 2308 in our tables, is fully annotated (NC_007618 and NC_007624; 2005). The second, designated in our tables as 2308 A, is a partially assembled and annotated draft assembly (GenBank wgs master record ACOR00000000; 2009), with nine contigs that span most of the 2308 genome.

**Table 1:** Insertions and deletions in *B. abortus* RB51 compared to *B. abortus* 2308 and 2308A

Mutation	Chrom.	2308 A Location or Gene	2308 Location or Gene	Annotation	Comments
12 bp deletion in RB51	I	BAAA_1000022	BAB1_0022	Hypothetical protein	Results in 4 aa deletion but no frameshift
8 bp deletion in RB51 *vs.* 2308 A	I	60776	60773	8 bp VNTR intergenic	
16 bp insertion in RB51 *vs.* 2308					
8 bp deletion in RB51	I	84770	84778	8 bp VNTR intergenic	
842 bp insertion in RB51	I	BAAA_1001034	BAB1_0999	*wboA*	Insertion of IS711 into *wboA* gene
8 bp insertion in RB51 *vs.* 2308 A	II	241110	241135	8 bp VNTR intergenic	
8 bp deletion in RB51 *vs.* 2308					
24 bp insertion in RB51 *vs.* 2308 (only)	II	N/A	72916	8 bp VNTR intergenic	RB51 has 5 copies; 2308 A has 5 copies; 2308 has 8 copies
8 bp deletion in RB51 *vs.* 2308	II	241118	241142	8 bp VNTR intergenic	RB51 has 5 copies; 2308 A has 4 copies; 2308 has 6 copies
8 bp insertion in RB51 *vs.* 2308 A					
162 bp deletion in RB51	II	BAAA_7000898	BAB2_0906	*narJ*	Disrupted gene

Across both RB51 chromosomes, we identified a number of insertion/deletion mutations as well as single-nucleotide polymorphisms (SNPs) as compared to the 2308 reference strains. First, insertion/deletion mutations in RB51, as compared to 2308 and/or 2308 A, are displayed in Table 1. There are only two genes with major disruptions in sequence, one each on Chromosome I and Chromosome II. On Chromosome I, *wboA*, a homolog of BAB1_0999, is interrupted by an IS711 insertion sequence element, as expected from previous sequence analysis of RB51 (as described above). In the nitrate reductase operon on Chromosome II, the *narJ* gene has a 162-bp deletion in the RB51 sequence, representing a novel finding. NarJ is a chaperone for a subunit of the nitrate reductase enzyme in *Brucella*. Nitrate reductase catalyzes the first step in denitrification, a process by which nitrate is converted via several processing steps into ammonia under anaerobic conditions. Expression of enzymes in the denitrification pathway in *Brucella* has been linked to virulence (Haine *et al.* 2006). Additionally, a smaller gene disruption is present on RB51 Chromosome I. An unidentified hypothetical gene, homologous to BAB1_0022 (which has a highly repetitive protein sequence), is disrupted by a 12-bp deletion in the RB51 genome. However, it does not cause a frameshift in the gene, and results in a four amino acid deletion in the protein product.

There are 38 SNPs (single nucleotide polymorphisms) in the RB51 Chromosome 1 genome as compared to strain 2308 (Table 2). Five nonsynonymous SNPs (highlighted with asterisks in the far left column) occur in annotated genes and are present in both the 2308 and 2308A genomes. The D to Y substitution in the beta subunit of DNA-directed RNA polymerase (orf01229, BAB1_1264, and BAAA_20000170) has previously been recognized as the basis for the rifampin resistance phenotype, but the phenotypic impacts of the rest of the SNPs in RB51 Chromosome I have not previously been characterized. Additionally, we identified multiple SNPs in the RB51 Chromosome II genome (Table 3); however, all of these SNPs were synonymous.

**Table 2:** Single nucleotide polymorphisms (SNPs)—Chromosome I

RB51		2308			2308 A			AA change (RB51/2308 A/2308)	Annotation
Residue	SNP	Residue	SNP	Locus ID	Contig-Residue	SNP	Locus ID		
277608	–	277613	G	intergenic	I-277620	—	intergenic	intergenic	—
*319721	A	319726	C	BAB1_0322	I-319733	C	BAAA_1000329	E/A/A	Secretion protein HlyD
464184	T	464188	—	intergenic	I-464196	T	intergenic	intergenic	—
*529443	A	529447	G	BAB1_0534	I-529455	G	BAAA_1000548	K/E/E	Polysaccharide biosynthesis protein CapD
542402	G	542406	T	BAB1_0550	I-542414	T	BAAA_1000566	V/G/G	Transposase, IS5/IS1182 family
542454	A	542458	C	BAB1_0551	I-542466	C	BAAA_1000567	synonymous	Transposase, IS4
542519	A	542523	G	BAB1_0551	I-542531	G	BAAA_1000567	synonymous	Transposase, IS4
542531	T	542535	G	BAB1_0551	I-542543	G	BAAA_1000567	synonymous	Transposase, IS4
551600	G	551604	T	BAB1_0562	I-551607	G	BAAA_1000581	Stop	Pseudogene region
*641998	C	642002	A	BAB1_0649*^a^*	I-642010	A	BAAA_1000669	T/K/K	Glutathione-S-transferase
754030	C	754034	T	BAB1_0773	I-754042	C	BAAA_1000793	A/A/V	Lipocalin-related protein Ppx/GppA phosphatase
755400	G	755404	T	BAB1_0774	I-755412	G	BAAA_1000794	A/A/E	Ribonuclease D
1116155	—	1115316	C	BAB1_1145	2-47001	—	BAAA_2000049	Frameshift in 2308	Hypothetical cytosolic protein
*1158574	T	1157735	G	BAB1_1186	2-89420	G	BAAA_2000088	M/L/L	Endoribonuclease L-PSP
1186080	G	1185241	A	BAB1_1216	2-116926	G	BAAA_2000120	synonymous	Tetracycline resistance protein TetB
1187024	G	1186185	A	intergenic	2-117870	G	BAAA_2000120	intergenic	—
1210357	T	1209518	G	BAB1_1242	2-141203	T	BAAA_2000148	K/K/Q	Ribosomal protein S14
1210423	G	1209584	A	BAB1_1243	2-141269	G	BAAA_2000149	L/L/F	Ribosomal protein L5
1210426	G	1209587	A	BAB1_1243	2-141272	G	BAAA_2000149	L/L/F	Ribosomal protein L5
1221625	A	1220786	C	intergenic	2-152471	A	BAAA_2000168	intergenic	Upstream (within 40 bp) of hypothetical protein BAB1_1262
1221646	A	1220806	—	intergenic	2-152492	A	BAAA_2000168	intergenic	Upstream (within 20 bp) of hypothetical protein BAB1_1262
*1228976	A	1228136	C	BAB1_1264	2-159822	C	BAAA_2000170	Y/D/D	RNA polymerase, beta subunit
1600922	C	1600081	—	BAB1_2222	2-531768	C	BAAA_2000572	23S rRNA	23S rRNA
1601072	—	1600232	C	BAB1_2222	2-531918	—	BAAA_2000572	23S rRNA	23S rRNA
1602012	A	1601172	—	BAB1_2222	2-532859	A	BAAA_2000572	23S rRNA	23S rRNA
1602031	G	1601190	T	BAB1_2222	2-532877	G	BAAA_2000572	23S rRNA	23S rRNA
1602033	T	1601192	A	BAB1_2222	2-532879	T	BAAA_2000572	23S rRNA	23S rRNA
1602053	A	1601212	G	BAB1_2222	2-532899	A	BAAA_2000572	23S rRNA	23S rRNA
1602075	C	1601238	T	BAB1_2222	2-532925	C	BAAA_2000572	23S rRNA	23S rRNA
1602425	C	1601584	T	BAB1_2222	2-533271	C	BAAA_2000572	23S rRNA	23S rRNA
1602864	C	1602023	A	BAB1_2222	2-533710	C	BAAA_2000572	23S rRNA	23S rRNA
1602883	C	1602042	A	BAB1_2222	2-533729	C	BAAA_2000572	23S rRNA	23S rRNA
1602902	C	1602061	A	BAB1_2222	2-533748	C	BAAA_2000572	23S rRNA	23S rRNA
1603421	—	1602581	G	BAB1_2222	2-534267	—	intergenic	23S rRNA	23S rRNA
1604211	—	1603372	T	intergenic	3-1252	—	intergenic	intergenic	—
1604550	—	1603712	G	BAB1_2225	3-1591	—	ribosomal	16S rRNA	16S rRNA
1605819	T	1604981	C	BAB1_2225	3-2860	T	ribosomal	16S rRNA	16S rRNA
1655204	T	1654366	T	intergenic	3-52245	C	intergenic	intergenic	—

a Note that the BAB1_0649 mutation was reported by us, based on the data generated in this study, by Gopaul *et al.* (2010) as a tool for molecular typing.

* Highlights nonsynonymous mutations of interest.

**Table 3:** Single nucleotide polymorphisms (SNPs)—Chromosome II

RB51		2308			2308A			AA change	Annotation
Residue	SNP	Residue	SNP	Locus ID	Residue	SNP	Locus ID		
292556	C	292588	T	BAB2_0299	292548	C	BAAA_7000304	synonymous	ABC sugar transporter, ATPase
381134	—	381167	C	intergenic	381147	—	intergenic	intergenic	—
492775	C	492808	T	BAB2_0500	492767	C	BAAA_7000503	synonymous	Glycine-betaine/L-proline ABC transporter ATPase; ProV
593384	A	593417	T	intergenic	593376	A	intergenic	intergenic	—
593387	G	593420	T	intergenic	593379	G	intergenic	intergenic	—
593400	—	593433	T	intergenic	593392	—	intergenic	intergenic	—
593408	C	593441	G	intergenic	593400	C	intergenic	intergenic	—
593410	G	593443	A	intergenic	593402	G	intergenic	intergenic	—
806397	A	806431	—	intergenic	806389	A	intergenic	intergenic	Upstream (within 50 bp) of BAB2_0821
866443	—	866476	C	intergenic	866435	—	intergenic	intergenic	—
1011554	A	1006508	—	intergenic	1011708	A	BAAA_7001034	intergenic	(Pseudogene region)
1011651	T	1006605	—	intergenic	1011805	T	BAAA_7001034	intergenic	—
1061165	—	1056118	A	23S rRNA	N/A	N/A	N/A	ribosomal	23S ribosomal RNA

Next, we compared our results to previously published reports of the RB51 genome sequence. In Ma *et al.* (2014), the RB51 genome was sequenced on an Illumina HiSeq platform, but the genome was not closed through the sequencing performed. While the specific locations of most SNPs were not elucidated in this report (Table S1), the authors did describe a SNP in the gene encoding the CapD protein that matches the SNP identified in our sequence. Therefore, there are multiple independent sources of confirmation of this mutation. A sequence has also been published to GenBank from the Broad Institute (NCBI reference NZ_AQIE00000000.1, not associated with a publication). Interestingly, we did not identify any of the non-synonymous SNPs described here in the NZ_AQIE00000000.1 sequence. Alignment of the Broad RB51 genome with our RB51 genome indicated that the expected *wboA* insertion, which is a defining characteristic of RB51, is not present in the Broad sequence (Figure S1).

Using gene locations on the BioCyc browser, we examined the location of each of the intergenic mutations, to consider the potential for each of these mutations to influence gene expression. Potential implications of the intergenic mutations are limited; mutations in RB51 observed within 50 bp of the upstream end of a predicted gene are indicated in Tables 2 and 3 (see Annotation column).

Notably, a previous study characterized the sequences of LPS synthesis genes in *B. abortus* RB51 in an attempt to understand the basis of the rough phenotype. Adone *et al.* (2011) amplified 21 genes known to be involved in LPS synthesis and compared their sequences to the *B. melitensis* 16M genome (Table S3). However, since RB51 is derived from *B. abortus* 2308, the 2308 parent strain is the proper point of comparison. Therefore, we analyzed the RB51 mutations identified by Adone *et al.* (2011) to compare them to the 2308 reference sequence. Consistent with our genome sequencing, only *wboA* (insertion) and *wbkD* (corresponds to *capD* in *B. abortus*; E559K mutation) carry mutations in RB51 as compared to *B. abortus* 2308; all other mutations identified by Adone *et al.* (Table S3) reflect sequence variation between *B. abortus* and *B. melitensis*.

### In silico evidence supports the importance of the CapD mutation to the rough phenotype of RB51

Based on its predicted function in bacterial envelope biosynthesis, the CapD mutation in RB51, relative to the 2308 sequence, is a promising candidate to explain the rough phenotype of the strain in conjunction with the *wboA* gene disruption. When the full-length CapD sequence (622 residues) was subjected to protein-protein BLAST against the Protein Data Bank (PDB) database, the top hit was the C-terminal domain of PgIF (E-value of 3e-82 for an alignment spanning 90% of residues of the PgIF C-terminal domain; Figure S2). PgIF is a sugar dehydratase in *Campylobacter jejuni*, for which the prokaryotic N-linked glycosylation pathway has been characterized in detail. More than sixty proteins in *C. jejuni* are glycosylated by a conserved heptasaccharide (Szymanski *et al.* 1999; Scott *et al.* 2011) that includes QuiNAc4NAc (2,4-diacetamido-2,4,6-trideoxy-α -D -glucopyranose) as one of its components. PgIF catalyzes the first step in the synthesis of QuiNAc4NAc, the dehydration of UDP-GlcNAc to UDP-2-acetamido-2,6-dideoxy-α-D-xylo-hexos-4-ulose. The enzymes involved in such 4,6-dehydratase events are known to belong to the NAD(H)- or NADP(H)-dependent short-chain dehydrogenase/reductase (SDR) superfamily (Kallberg *et al.* 2002a; Kallberg *et al.* 2002b; Kavanagh *et al.* 2008; Kallberg *et al.* 2010). In these enzymes, the dehydration step is flanked by dehydrogenation/ hydrogenation steps involving NAD(P).

PglF is a NAD^+^-dependent membrane associated protein (590 residues) composed of three domains: an N-terminal motif containing four transmembrane regions, a linker section of unknown function, and a C-terminal catalytic domain (Schoenhofen *et al.* 2006). The structure of the C-terminal domain of PglF (Ala 244 to Lys 587) has been solved by x-ray crystallography (Protein Data Bank ID 5BJU; Riegert *et al.* 2017). A BLAST alignment performed between the full-length sequences of CapD and PgIF (Figure S4) yields a highly significant E-value (7e-97), indicating that the homology between CapD and PgIF extends to the entire protein. Moreover, as in PgIF, CapD is also predicted to contain four transmembrane helices in the N-terminal region (data not shown).

Therefore, we utilized the PglF structure to model the impact of the CapD mutation, using the crystal structure of the C-terminal domain of PglF as a template to construct a model of the C-terminal domain (residues 261 – 585) of CapD. In the structural superposition (Figure S3), the key active site residues of PgIF superposed with residues of CapD (in parentheses) as follows: 395 Thr (413 Ser), 396 Asp (414 Asp), 397 Lys (415 Lys). Accordingly, two of the active site residues are strictly conserved in CapD, while the third is a conservative Thr to Ser substitution. Thr 395 Ser PgIF mutants have been shown to be fully active (Riegert *et al.* 2017). Thus, the “CapD-C-term” model provides strong evidence that CapD acts as a sugar 2,4-dehydratase. PgIF belongs to a subclass of the SDR superfamily in which the Tyr in the YXXXK signature sequence (Kallberg *et al.* 2002) has been replaced by a Met. The structural alignment also suggests that CapD belongs to this same subclass (Met 423 of CapD superposes with Met 405 of PgIF).

**Figure 1:**
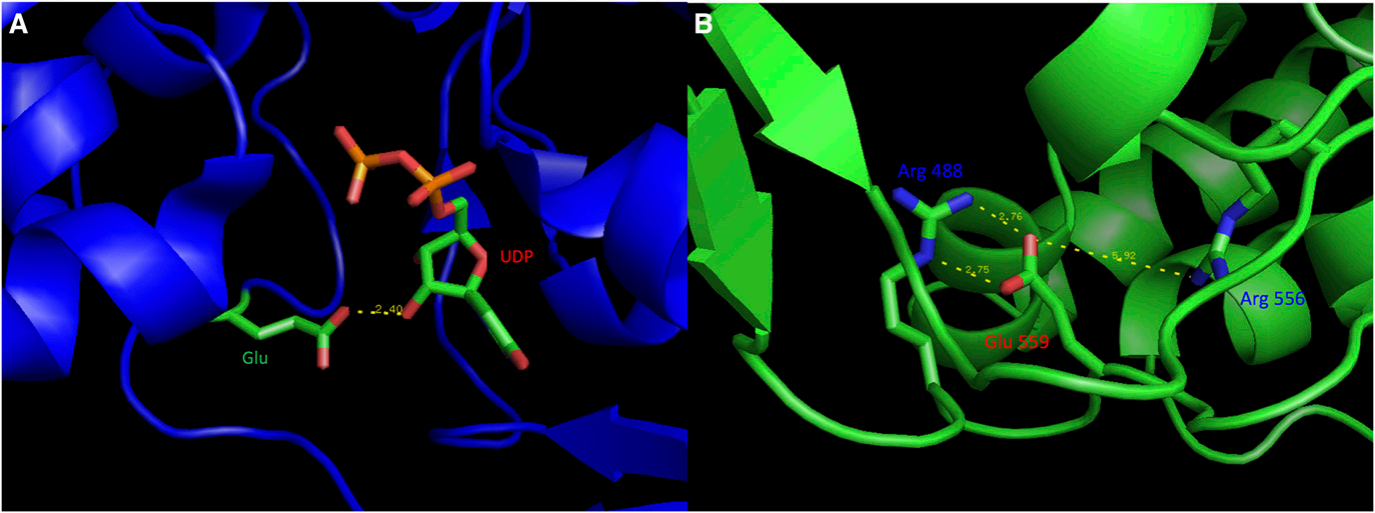
Glu 559 of CapD is predicted to be directly linked to enzyme activity. (A). In the CapD homolog PgIF (PDB ID 5BJU), the corresponding glutamate, Glu 526, makes hydrogen bonding interactions with the C-2 hydroxyl group of the ribose moiety in UDP. (B). In the model for the C-terminal domain of CapD (CapD-C-term), Glu 559 participates in salt bridge type interactions with Arg 488 and Arg 556 (similar interactions are seen in PgIF). The Glu 559 Lys mutation in strain RB51 would be predicted to create repulsive interactions between positively charged side chains, disrupting the putative binding site for UDP.

Structural superposition of the CapD-C-term model with the C-terminal domain of PgIF (Figure S2) shows Glu 559 of CapD superposing with Glu 526 of PgIF. Importantly, Glu 526 is part of the binding pocket for one of the substrates of PgIF, uridine-5′-diphosphate (UDP); Glu 526 specifically forms hydrogen bonding interactions with the ribose moiety of UDP (Figure 1A). In addition, Glu 526 makes salt bridge-type interactions with two arginine residues, stabilizing the binding pocket for UDP. The model for the C-terminal domain of CapD (CapD-C-term) does not contain UDP, but the close superposition of the C-terminal domains suggests that Glu 559 of CapD could make similar hydrogen bonding interactions with a putative UDP substrate. Further, in CapD-C-term, Glu 559 makes stabilizing salt bridge interactions with two Arg residues (Figure 1B) similar to that observed for Glu 257 in PgIF (as discussed above).

Finally, additional evidence for the functional importance of Glu 559 of CapD comes from mutational studies previously performed on the related SDR superfamily member CapE of *S. aureus*. The structural superposition between CapD-C-term and the crystal structure of CapE (Figure S5) indicates that Glu 257 of CapE superposes with Glu 559 of CapD. Mutating Glu 257 of CapE to Ala leads to a loss of activity (Miyafusa *et al.* 2013), suggesting that the Glu 559 Lys mutation observed in RB51 CapD would also lead to a similar loss of activity, and impaired glycosylation of proteins in the RB51 strain.

## Discussion

The presentation of a complete RB51 genome comparison to two published 2308 sequences defines a set of mutations for future genetic analysis of the properties of *B. abortus* and/or *B. melitensis* strains modified at one or more of these genetic locations. Our data not only confirm the presence of the CapD mutation in RB51, but utilize *in silico* modeling to provide evidence supporting the hypothesis that this mutation is the second key RB51 mutation contributing to a rough strain phenotype. Previous studies have demonstrated that *B. melitensis* strains carrying a *wboA* mutation are attenuated and have increased vaccination efficacy (Li *et al.* 2015); therefore, the *Brucella capD* gene is a candidate for future mutation in vaccine development.

Additionally, the other mutations identified in RB51 chromosome II serve as the basis for future wet lab studies of the influence of these genes on *Brucella* phenotypes. Recently, Herrou *et al.* (2019) reported on the first structural and functional characterization of the protein product of BAB1_1186, which they have named EipA; EipA is conserved in *Brucella* and is proposed to be involved in cell envelope homeostasis. Deletion of *eipA* in *B. abortus* revealed that the protein is not required for initial spleen colonization in mice, but is involved in persistence in the spleen (Herrou *et al.* 2019). BLAST analysis reveals that the L → M mutation in EipA observed in RB51 is not present in other *Brucella* species; this biochemical change has the potential to influence function as a destabilizing mutation (Lipscomb *et al.* 1998), and is a candidate for future investigation. Finally, the disruption of *narJ* is of phenotypic interest, due to the potential to compromise survival of the vaccine strain in macrophages. Introduction of the RB51 complement of genetic mutations, or a subset of these, into *B. melitensis* could provide the basis for a new attenuated vaccine candidate in this closely related, but challenging, species.
